# Sinoatrial node pacemaker cells: cardiomyocyte- or neuron-like cells?

**DOI:** 10.1007/s13238-021-00827-w

**Published:** 2021-02-06

**Authors:** Bin Zhou

**Affiliations:** State Key Laboratory of Cell Biology, Shanghai Institute of Biochemistry and Cell Biology, Center for Excellence in Molecular Cell Science, University of Chinese Academy of Sciences, Chinese Academy of Sciences, Shanghai, 200031 China

The sinoatrial node (SAN) is the headquarter of heartbeat throughout our lifetime (Lakatta et al., 2010; Cingolani et al., 2018; Peters et al., 2020). Every beat of the heart is triggered by a bioelectric pulse spontaneously released by SAN pacemaker cells (SANPCs) (Yaniv et al., 2014; Yavari et al., 2017). In adult human heart, the SAN is a crescent-shaped structure of 1–2 cm long and 0.5 cm wide, which is located at the junction of the superior vena cava and the right atrium and lies along the sulcus terminalis (John et al., 2016). However, the nature of SANPCs remains incompletely known. In general, SANPCs have long been considered as specialized cardiomyocytes (Van Eif et al., 2018; Linscheid et al., 2019; Galang et al., 2020; ). However, SANPCs do not have myofibril and T-tube, thus not sharing the contractility property of cardiomyocytes (Satoh, 2003; Protze et al., 2017). Interestingly, SANPCs share some electrophysiological characteristics with neurons: excitability and conductivity. In addition, SANPCs have their intrinsic autonomic rhythm, while neurons also possess the intrinsic ability to generate spontaneous electrical impulses (Lisman et al., 2018). Whether SANPCs are neuron-like cells that reside in the heart remains enigmatic in the field.

In this issue of Protein & Cell, Chen and his colleagues demonstrated that, by single-cell transcriptome analysis, SANPCs co-clustered with neurons of visual cortex. SANPCs expressed cell markers of glutamatergic neurons and contained key elements of glutamatergic neurotransmitter system, including glutamate synthesis pathway, ionotropic glutamate receptors, metabotropic glutamate receptors, and glutamate transporters. Interventions targeting this glutamatergic neurotransmitter system had an extraordinary effect on the spontaneous pacing frequency of isolated SAN tissues and spontaneous Ca^2+^ transients frequency in single SANPC. This elegant work reveals for the first time that SANPCs are glutamatergic neuron-like cells in the heart, shedding new light on our understanding of the fundamental biology of SAN. This finding provides a novel intrinsic regulation module of heart rhythm and indicates an entirely new concept of pharmaceutical development for prevention and treatment of sinus tachycardia or sinus bradycardia.

It has been known that the SAN is regulated by the autonomic nervous system, which includes the sympathetic and parasympathetic nerves (Shen et al., 2014; Herring et al., 2019). Excitement of sympathetic nerves leads to sinus tachycardia, whereas excitement of parasympathetic nerves leads to sinus bradycardia. Based on the expression of adrenergic and cholinergic receptors in the SAN, the drugs targeting these receptors are used to treat sinus arrhythmias clinically (Kusumoto et al., 2019; Mesirca et al., 2020). Does it mean that the glutamatergic neurotransmitter system in SAN is another regulatory system, just like the autonomic nervous system? Actually, the observed glutamatergic neurotransmitter system in SANPCs is different from that of autonomic nervous system. For the SANPCs, the autonomic nervous system belongs to foreign innervation, while the glutamatergic neurotransmitter system is the endogenous regulatory system. Professor Chen and his colleagues revealed that SANPCs possessed this inherent heart rate regulation module, i.e., the glutamatergic neurotransmitter system, and elucidation of its presence provides an important new direction for future exploration and further investigation.

As an intrinsic regulation module of the SAN autonomic rhythm, what is the significance of the glutamatergic neurotransmitter system for the prevention and treatment of arrhythmia? SAN dysfunction can lead to bradycardia, cardiac arrest, syncope or even sudden cardiac death (De Ponti et al., 2018; Sharma et al., 2018). Unfortunately, few treatment options are available for SAN related disorders due to lack of comprehensive understanding of SAN. The newly identified glutamatergic neurotransmitter system inherent in the SANPCs may serve as a new intervention target for SAN diseases. Moreover, considering that SANPC-like cells are present in atrioventricular nodes and Purkinje fibers as well, it is possible that the glutamatergic neurotransmitter system may also be a potential therapeutic target for the treatment of atrioventricular node or Purkinje fibers dysfunction.

In addition, the authors revealed that embryonic SANPCs also co-clustered with glutamatergic neurons, suggesting that embryonic SANPCs have already shared some cell property and features of glutamatergic neurons. However, it seemed that the co-clustering pattern was not identical between embryonic and adulthood, which indicates the maturity of SANPCs from embryonic to adult stage. It is unclear when this glutamatergic neuron property first appears and whether this property is related to the functional maturation and specialization of SANPCs. Interestingly, the SANPCs exhibited obvious heterogeneity when co-clustering with neurons both at embryonic and adulthood, revealing the complexity and diversity of SANPCs at different stages.

The study not only enhances our understanding of the molecular and cellular nature of SAN in the regulation of heart rhythm, but also facilitates the development of potential new strategies for the prevention and treatment of cardiac arrhythmias. The findings of Chen’s group provide a new angle on the research of cardiac conduction systems and represent a conceptual breakthrough in this field.
